# Circulating Tumor Cells in Pancreatic Cancer: Current Perspectives

**DOI:** 10.3390/cancers11111659

**Published:** 2019-10-26

**Authors:** Verena Martini, Sylvia Timme-Bronsert, Stefan Fichtner-Feigl, Jens Hoeppner, Birte Kulemann

**Affiliations:** 1 Department of General and Visceral Surgery, Medical Center-University of Freiburg, Faculty of Medicine, University of Freiburg, 79106 Freiburg, Germany; verena.martini@uniklinik-freiburg.de (V.M.); stefan.fichtner@uniklinik-freiburg.de (S.F.-F.); jens.hoeppner@uniklinik-freiburg.de (J.H.); 2 Institute for Surgical Pathology, Medical Center-University of Freiburg, Faculty of Medicine, University of Freiburg, 79106 Freiburg, Germany; sylvia.timme@uniklinik-freiburg.de; 3 Comprehensive Cancer Center Freiburg-CCCF, Medical Center-University of Freiburg, 79106 Freiburg, Germany

**Keywords:** pancreatic ductal adenocarcinoma, circulating tumor cells, liquid biopsy, biomarker, overall survival

## Abstract

Pancreatic cancer is the fourth leading cause of cancer-related death in the USA and Europe; early symptoms and screenings are lacking, and it is usually diagnosed late with a poor prognosis. Circulating tumor cells (CTCs) have been promising new biomarkers in solid tumors. In the last twenty years (1999–2019), 140 articles have contained the key words “Circulating tumor cells, pancreatic cancer, prognosis and diagnosis.” Articles were evaluated for the use of CTCs as prognostic markers and their correlation to survival in pancreatic ductal adenocarcinoma (PDAC). In the final selected 17 articles, the CTC detection rate varied greatly between different enrichment methodologies and ranged from 11% to 92%; the majority of studies used the antigen-dependent CellSearch^©^ system for CTC detection. Fifteen of the reviewed studies showed a correlation between CTC presence and a worse overall survival. The heterogeneity of CTC-detection methods and the lack of uniform results hinder a comparison of the evaluated studies. However, CTCs can be detected in pancreatic cancer and harbor a hope to serve as an early detection tool. Larger studies are needed to corroborate CTCs as valid biomarkers in pancreatic cancer.

## 1. Introduction

Pancreatic ductal adenocarcinoma (PDAC) is the fourth leading cause of cancer-related death in the United States and Europe [1,2]. Its incidence almost equals mortality with a five-year survival rate of <6% [2], and it will likely be the second leading cause of cancer-related deaths in 2030, exceeding mortality from breast and colorectal cancer [3]. The disease is often detected in metastatic stages, reflecting its late diagnosis and aggressive biology. Early symptoms are rare and most patients initially present with mild symptoms such as abdominal discomfort, back pain, tiredness and weight loss. Distant organ metastases are the final result of hematogenous cancer cell spread, with which about 80% of patients are diagnosed; no sufficient screening regimen is presently used for the early detection of PDAC.

Currently, most treatment decisions are based on the tumor stage estimated by cross-sectional imaging and endoscopic tumor biopsy. Computed tomography (CT) and magnet resonance imaging (MRI) are widely applied, but MRI has the higher diagnostic sensitivity and accuracy [4]. About one-fifth of the patients are “under-staged” since metastatic disease is often only visible upon operative exploration [4].

To date, only serum CA19.9 is routinely used as a non-invasive blood-based biomarker in PDAC. It does not, however, function as diagnostic tool alone—but high CA19.9 levels often indicate advanced tumors [5]. The concern regarding this biomarker is that an elevated expression of CA19.9 can be found in various benign (pancreatitis, acute cholangitis and cirrhosis) and malignant (colorectal cancer, gastric cancer, bladder cancer and uterine squamous cell carcinoma) diseases in addition to PDAC, resulting in its non-specificity. Additionally, about 10% of the Caucasian population do not express CA19.9, and only 65% of patients with resectable PDAC have elevated levels of CA19.9 [6]. CEA also serves as a biomarker, though it has some of the shortcomings of CA19.9 and also lacks specificity.

Other blood-based biomarkers are not routinely used or have not yet entered clinical practice. Liquid biopsies, such as circulating tumor DNA (ctDNA), exosomes, plasma proteomics and circulating tumor cells (CTCs) have now been investigated for over a decade and have presented encouraging results, which are partly discussed in this review; however, clear clinical recommendations for patients with PDAC are still missing.

The routine treatment of resectable PDAC includes complete tumor resection followed by heterogeneous chemotherapy regimens. Gemcitabine is routinely applied as a first-line therapy with a survival benefit of several months [7]. Historically, neoadjuvant treatment regimens have shown outcome benefits. Without randomized trials, however, physicians stick to upfront surgery in resectable cases [8]. Some new adjuvant chemotherapeutic protocols have lately come into clinical practice. Gemcitabine, FOLFIRINOX and paclitaxel have been applied as part of clinical studies, and the combination of nab-paclitaxel and gemcitabine has recently shown an extended overall survival in metastatic pancreatic carcinoma compared to gemcitabine alone [9]. The same results have been documented for gemcitabine and FOLFIRINOX [10] with higher toxicity and more adverse events in the FOLFIRINOX group in the metastatic stage. The current PANACHE01 trial evaluates the efficacy of two neoadjuvant chemotherapeutic regimens with FOLFOX and mFOLFIRINOX compared to standard treatment with surgery followed by adjuvant gemcitabine in resectable PDAC [11].

The limited diagnostic abilities, as well as the complex and narrow treatment options highlight the need for a prognostic and predictive biomarker. A prognostic biomarker can provide insight into a patient’s outcome, independent of the treatment received. This biomarker can give an estimation of survival, time to disease progression, or type of progression. Differently, a predictive biomarker is able to identify a subset of patients who would benefit from specific cancer treatment and hence can guide treatment decisions. An ideal biomarker is non-invasive, repeatedly accessible, reasonably-priced, and simple to analyze.

In this review, we give an overview of the current literature on CTCs in pancreatic cancer in a clinical context. We focus on studies that evaluate the clinical applications of CTCs in patients with PDAC. We also discuss the problems of inhomogeneous CTC isolation and analysis, and we highlight potential research perspectives.

## 2. Diagnosis and Etiology of Pancreatic Cancer

Patients with pancreatic cancer often present with unspecific symptoms such as weight loss, back pain, abdominal pain, and fatigue. Cross-sectional imaging, endoscopy, serum biomarkers, and histological biopsies are currently potential tools used in clinical routines to diagnose pancreatic cancer [12,13]. When imaging is not conclusive, and before oncologic treatment, endoscopic ultrasound techniques are combined with fine-needle aspiration in order to obtain a biopsy and secure the tumor diagnosis. Due to the anatomical position of the pancreas, this procedure is technically challenging and carries a high level of complication. Additionally, false negative results occur in many cases due to the cannulation of stromal cells which represent the majority of the tumor mass [14]. Repeated invasive biopsies are the consequence and harbor a risk for tumor spread and post-interventional complications. Some cases require laparoscopic- or even open-surgery to obtain a tumor specimen to confirm the diagnosis of pancreatic cancer. To date, no specific screening tests are available for pancreatic cancer.

The causes of pancreatic cancer (PC) are multifactorial and diverse. High-risk groups are patients with a positive family history of PC, hereditary PC, familial atypical multiple mole melanoma (p16 mutation), cystic fibrosis, Peutz–Jeghers syndrome, Lynch syndrome and HBOC (BRCA1/BRCA2 mutation). Risk factors for the development of PC are high age, male gender, obesity, smoking, pancreatitis, and diabetes mellitus. New-onset diabetes mellitus is considered a risk for PC, but whether it is the cause or effect of PC is still unclear [4,7,15].

PDAC is characterized by multiple genetic variants which have been implicated in tumor formation and progression. Point mutations in the *Kirsten rat sarcoma viral oncogene homolog* (*KRAS*) gene are present in over 90% of tumors [7,16,17,18] and are thought to be an early event in tumorigenesis. Other driver mutations like TP53, p16 and SMAD4 are also commonly found in PDAC specimens [15,16]. More recently, the theory of a “three-step procedure” of carcinogenesis was introduced for PDAC. These steps are: First, the initiation of the tumor by the acquisition of a driver gene mutation in a cell of origin; second, the clonal expansion of the mutation-carrying cell into a multicellular neoplasm; and third, the introduction of the neoplastic cells into both local and distant microenvironments [19,20]. The first step includes the development of pancreatic intraepithelial neoplasms (PanINs) harboring *KRAS* mutations [21] that function as precursor lesions. In other words: Pancreatic tumorigenesis most likely takes years, which offers a long window of time for tumor diagnosis and early intervention.

## 3. Circulating Tumor Cells in Pancreatic Cancer

CTCs were first pointed out 1896 by the Australian Thomas Ashworth, who described the microscopic observation of CTCs in the blood of a patient with metastatic breast cancer. He postulated: “Cells identical with those of the cancer itself being seen in the blood may tend to throw some light upon the mode of origin of multiple tumors existing in the same person” [22]. It took almost 150 years after this discovery to establish a routine identification of CTCs.

CTCs in the blood stream are thought to represent disseminated tumor cells that have detached from the primary lesion and that are undetectable by clinical imaging and inaccessible to excision. CTCs are thought to be the basis for distant metastasis [23]. Their rarity among billions of blood cells explains the challenge of specific identification and isolation. While CTCs have been extensively studied in other neoplasms, their significance in PDAC at various stages is not completely understood. There is, however, emerging evidence that CTCs may also serve as a valuable tool for the outcome prediction and understanding of tumor biology in PDAC [24,25,26,27].

CTCs have been found in all stages of PDAC, starting even with precursor lesions, as described in a subsequent section of this paper [23,24]. Circulating epithelial cells (CECs) have also been found in benign pancreatic lesions such as pancreatitis [23,28,29]; to date, the significance of these finding remains unclear. The detection rate of CTCs as well as the methods to detect them vary greatly. Detection rates have been described from 11% [30] up to 92% with isolation by size [31] or a NanoVelcro assay [15]. To your knowledge, however, it has not been reported that CTCs and CECs can be found in healthy individuals.

Its central location surrounded by important structures makes biopsies challenging and bears the risk of complications. Even the routine and relatively safe endoscopic ultrasound (EUS)-guided fine-needle aspiration (FNA) sampling, the “gold standard” for PDAC diagnosis, is frustrating in 15%–20% of the cases [32,33]. Additionally, the limited number of cells does not always allow for the complete phenotypic and genetic profiling of the retrieved cells, leading to diagnostic limitations. Due to an extensive proportion of stromal cells in tumors, false negative sampling is also possible. Peripheral blood samples can be easily taken at one or multiple time points over the course of treatment without any harm for the patient, and there has been emerging evidence that liquid biopsy may serve as a surrogate for tumor tissues [34]. However, future studies are needed since tumor heterogeneity is a phenomenon that has only been included as a relevant factor in CTC analysis within the last decade [35,36,37].

**Figure 1:**
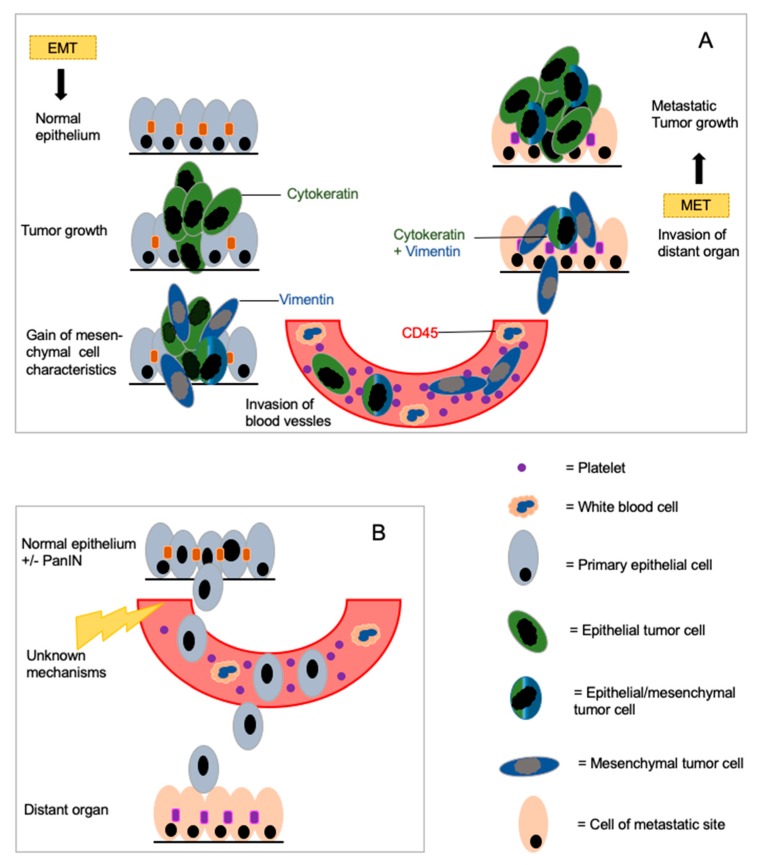
Schematic display of epithelial–mesenchymal transition (EMT) and mesenchymal–epithelial transition (MET) in pancreatic cancer development. Circulating epithelial, circulating mesenchymal and circulating epithelial/mesenchymal cells are displayed to show the heterogeneity of circulating tumor cells (CTCs) (**A**). (**B**) shows the concept of early dissemination before malignancy. Mechanisms involved in this process are widely unknown and part of current research.

One important point of CTC analysis in PDAC is the concept of epithelial–mesenchymal transition (EMT). This is a biological process in which polarized cells that are usually in contact with the basement membrane undergo multiple biochemical changes and gain mesenchymal properties. This leads to an enhanced migratory capacity, invasiveness and elevated resistance to apoptosis. Cells can detach from the primary lesion and enter into the bloodstream (Figure 1A). At a distant site, they can undergo the reverse process, the mesenchymal–epithelial transition (MET) to induce a new metastasis [38]. EMT is involved in embryo formation and organogenesis (type 1), in wound healing (type 2), and in the formation of neoplastic cells and metastases (type 3) [39].

EMC in pancreatic cancer seems to be associated with portal vein invasion and lymph node metastasis. Furthermore, premalignant pancreatic lesions (IPMN—intraductal papillary mucinous neoplasm—borderline and carcinoma in situ) also undergo EMT. CTCs are thought to be heterogeneous groups of cells with varying phenotypic and genotypic properties. In lung cancer [37] and breast cancer [40], new studies on single CTC have shown substantial inter- and intra-patient heterogeneity: Different types of CTCs have been found among different patients but also within one patient. Our own studies have also shown a heterogeneous group of cells in the blood with different staining and *KRAS* mutational properties [16,31]. CTCs with both epithelial and mesenchymal markers have been found. The presence of the mesenchymal EMT marker ZEB1 (Zinc finger homebox 1) and the expression of epithelial CK (cytokeratin) have shown no statistically significant impact on survival in patients with PDAC [16]. In contrast, Rhim et al. were able to show that EMT-transformed cells have tumor initiating properties [23].

### 3.1. Methods of CTC Isolation and Detection

CTCs are very rare. With one cell in 10^6^–10^8^ leukocytes in 1 mL of whole blood, their detection is a challenge, especially in PDAC due to their low rate of occurrence in comparison to other tumor entities. Thus, CTC isolation is generally a two-step procedure. Step one is CTC enrichment followed by step two, CTC detection.

CTC enrichment techniques are as follows:

- Physical/biological systems isolate CTCs on the basis of electric charge or cell size. The enrichment relies on the fact that CTCs have a higher density, a different electric charge, a different motility, and a larger size than normal blood components. They can thus be isolated with dedicated devices (microfilters, microfluidic chips, electric separation, etc.).
- Surface antibody–based enrichment can be used as “positive” or “negative” enrichment. Positive selection relies on antibodies directed against the surface markers of the CTCs. Common CTC isolation techniques use the epithelial cell adhesion molecule (EpCAM) for positive selection. To eliminate normal blood components, the negative selection systems deplete leukocytes from the specimens. They use CD45 antibodies to bind the blood components and to isolate the CD45-negative CTCs.

The second step—the detection of the enriched CTCs—can also be performed with various approaches.

- Morphological examination supported by immunocytological staining for cancer-specific antibodies is the gold standard for CTC detection and definition. This can be performed after physical enrichment but can also be performed in part with surface-antibody based methods, even though the cytological evaluation is not as good as for regular cytological specimens.
- mRNA analysis for epithelial or pancreatic markers without morphologic control has been performed [41,42,43] and has shown interesting results in PDAC.
- Mutational analysis of the DNA of enriched cells for tumor-specific mutations in PDAC—typically *KRAS*. This also allows for comparison of the mutational landscape of the CTC with the primary tumor with a high specificity. Our own results showed a heterogeneous picture of the CTC in comparison with the primary tumor; we had about 40% discordant “CTC-primary tumor pairs” [31]. Ankeny et al. found a 100% concordance in five pairs tested [25].

Technologies based on physical properties such as ScreenCell, ISET^®^ (isolation by size of epithelial tumor cells), ApoStream™, ScreenCell^®^, ClearCell^®^ FX System, and density or gradient centrifugation are used to enrich and detect CTCs. The size-based or fluidic-based selection strategies use the fact that CTCs are physiologically different and notably larger in size than normal blood components [44]. Small CTCs that are cytokeratin-positive, CD45-negative, and morphologically resemble white blood cells are, however, not captured by this technique.

The surface antibody-based technique by CellSearch^©^ (Menarini Biosystems, IT) has been approved by the FDA for CTC detection in breast, colorectal, and prostate cancer, and the authors refer to this technique as the “gold standard” for CTC isolation [45]. The CellSearch^©^ system captures CTCs with the use of the epithelial cell adhesion molecule (EpCAM) followed by the characterization of the captured CTC with cytokeratin positivity and CD45 negativity to exclude white blood cells. Recent studies, however, have shown that the EpCAM-based strategy fails to detect CTCs with low EpCAM expression, since CTCs may lose their epithelial antigens during the EMT process. Moreover, these EpCAM-negative CTCs seem to be an aggressive and invasive subtype [36,46,47]. Cells that express the leukocyte antigen CD45 are not classified as CTCs but as regular blood components by the CellSearch^©^ technique. However, this ignores the CTC–blood cell interaction that leads to “CD45-positive” clusters, some of which may contain CTCs. A recent study also investigated the cell–cell interaction of CTCs and neutrophil granulocytes within the blood stream. The authors found cell cycle genes upregulated in CTC-granulocyte clusters and concluded that the “association between neutrophils and CTCs drives cell cycle progression within the bloodstream and expands the metastatic potential of CTCs, providing a rationale for targeting this interaction in the treatment of breast cancer.” [48]. In this manner, EpCAM-based methods may underestimate those CTCs that are highly aggressive and invasive [14].

In summary, there is a broad heterogeneity in the detection methods of CTCs, none of which are perfect. The wide variety of results reported in the literature makes a comparison barely possible. Future studies should combine different isolation techniques to reach a better comparability until an innovative strategy has been developed that combines immunological, physiological and genetic analyses in one device. A single-cell CTC analysis will help us to identify the biologically-relevant fraction of CTCs that cause metastases.

### 3.2. Clinical Utility of CTCs

#### 3.2.1. Differential Diagnosis and Early Detection

No standardized screening tool exists for PDAC beyond imaging; thus, diagnosis and differential diagnosis rely on cross-sectional imaging and EUS accompanied/confirmed by cytologic specimens. Other preneoplastic lesions (such as IPMN) or benign lesions (such as chronic pancreatitis) have to be distinguished from cancer by imaging and EUS-FNA. An over-interpretation of benign lesions is as much a reality as the false “tumor-negative” classification of a pancreatic mass. A diagnostic tool to screen and to differentiate pancreatic cancer from other pancreatic lesions would be extremely helpful. CTCs or CECs have been evaluated in benign diseases as well as in precursor lesions in pilot studies. A study by Rhim et al. found CECs in 33% of patients with IPMN but without a malignant tumor. In PDAC, they found these cells in 73% of the cases [49]. The same group found pancreatic CECs in premalignant stages in a mouse model before frank malignancy occurred. The cells entered the blood stream much earlier than expected (Figure 1B). Additionally, treatment regimens with dexamethasone led to the almost complete disappearance of CECs in these mice, suggesting that inflammatory stroma facilitate EMT and allow early dissemination [23]. These findings in mice and humans are novel and open a variety of therapeutic opportunities for high-risk patients. The authors stated that “...if these cells represent the earliest forms of cancer, we predict that they would contain a complement of somatic mutations associated with PDAC.” Studies evaluating the genetic landscape of these CECs and long-term studies to evaluate the development of PDAC in these patients are currently being performed [49].

Cauley et al. published the results of the CEC-cytology of 179 patients with a variety of pancreatic lesions such as chronic pancreatitis, IPMN, PDAC and neuroendocrine tumors [29]. Interestingly, rates of CEC identification were similar in patients with benign, premalignant, and malignant lesions, and CEC findings in PDAC patients were not associated with a poor prognosis in a follow-up of one year. A more detailed morphologic analysis of these cells revealed a similar cytological appearance in patients with benign and malignant disease [28]. Immunophenotypic and genetic information is lacking in these studies; neither mutational analysis, proliferation analysis (ki67) nor phenotypic analysis have been performed. In summary, there are circulating epithelial cells in transit in patients with premalignant and benign diseases. This leads to the potential for tumor detection in premalignant stages. However, clinical utility remains obscure and needs further investigation.

To our knowledge, there have thus far no publications regarding the utility of circulating tumor cells for early detection and screening in PDAC patients. Circulating tumor DNA (ctDNA), not particularly a part of this review, has, however, gained recent focus in order to improve this field. A study by Cohen et al. found a combination of well-thresholded plasma proteins and a ctDNA analysis for *KRAS* mutations very useful for the earlier detection of PDAC. The sensitivity of this combination test was 64%, with 99.5% specificity [50].

Nevertheless, CTCs harbor the potential for earlier tumor detection and future, large-scale trials are needed. Two relevant studies are registered in the study registry clinicaltrials.gov: One from Rouen, France (NCT02072616) explores CTCs for improvements in diagnostic testing; the other study from Missouri, Columbia (NCT03551951) explores liquid biopsy markers including CTCs in various solid tumors. To our knowledge, there is no large-scale (>1000 patients) CTC screening study for PDAC in preparation.

#### 3.2.2. CTC as Prognostic Marker

CTCs have been shown to serve as prognostic markers in several pilot studies (Table 1). Conventional prognostic factors such as tumor size, nodal status, and perineural invasion can be evaluated only after tumor resection and mostly confirm a poor prognosis. This is different with CTCs, because they offer a window into cancer development and progression in a blood sample. A meta-analysis from 2014 included nine cohort studies with over 600 PDAC patients (UICC stage I–IV). It showed that the patients with positive CTCs (43%) had a poorer progression-free survival (PFS) (*p* < 0.001) and overall survival (OS; *p* < 0.001) than CTC-negative patients, suggesting that CTCs may be promising biomarkers for the prognosis of PDAC [51]. Still, results have greatly varied and are dependent on the isolation technique used and the patient population. Bidard et al. investigated patients with locally advanced PDAC and found 11% of CTC-positive patients with CellSearch^®^. This finding was, however, clinically relevant; patients with CTCs had a worse OS [30]. Others using the CellSearch^®^ technique also reported a relatively low CTC positivity [52]. Studies which have used antibody-independent CTC isolation have usually had more CTC-positive patients (86%–92%) [24,27,53], but some studies have reported only 34% of positive patients after density gradient separation.

In a recent study of 50 patients with PDAC, Poruk et al. evaluated CTCs isolated with the label-free ISET^®^ device and stained for cytokeratin and the mesenchymal-marker vimentin. The presence of cytokeratin-positive CTCs was shown to be a significant independent predictor of survival by univariate and multivariable analyses after accounting for other prognostic factors. The detection of CTC co-expressing markers, vimentin and cytokeratin, was shown to be predictive for tumor relapse. Patients with vimentin-positive and cytokeratin-positive CTCs had an earlier recurrence than patients without CTCs [54]. The same group evaluated the markers for tumor initiating cells (TIC) and stemness in CTCs found in patients with PDAC (cytokeratin, CD133, CD44, and ALDH). These markers were explored as prognostic factors in a group of 60 patients. They found that ALDH-positive CTCs and triple-positive CTCs were significantly associated with worse survival by univariate analysis, and ALDH-positive CTCs, triple-positive CTCs, and dual cytokeratin- and CD133-positive CTCs were not only independent predictors of tumor recurrence but were also associated with shorter disease-free survival [55].

A group from Baltimore provided the thus far largest study on CTC dynamics over treatment. In the CLUSTER study, multiple blood samples from 200 patients with presumed PDAC were prospectively collected and evaluated for CTCs with the label-free ISET^®^ device over the course of treatment. Out of all the CTCs (tCTC), the authors identified two major groups of CTCs: eCTC, with epithelial phenotype (cytokeratin+ and vimentin−) and mCTC with a mixed epithelial/mesenchymal phenotype (cytokeratin+ and vimentin+). Additionally, they performed a ‘‘cocktail’’ stain to separate different white blood cell (WBC) populations (anti-CD45, CD11b, CD14, and CD34). Of the 136 resected patients, 56 had to undergo neoadjuvant chemotherapy. These patients showed significantly fewer CTCs of all subtypes; surgery let the CTC counts drop severely. It was striking that preoperative numbers of all CTC subpopulations were the only predictors of early recurrence within 12 months after surgery in the pretreated and the primarily resected patients. OS was significantly longer in the pretreated patients without CTCs before therapy.

In the chemo-naïve group, CTCs were not associated with a worse overall survival. The authors also found an increase of CTCs before recurrence occurred and developed a risk assessment score based on the difference of tCTC increase. This score accurately identified disease recurrence within the next two months, with an accuracy 75% [61]. In summary, there is emerging evidence for (subsets of) CTCs as prognostic markers and clinically relevant tools. All studies mentioned above are displayed in Table 1.

#### 3.2.3. CTC as Predictive Marker

A recent meta-analysis on intensified chemotherapy in PDAC revealed that only about 20–30% of the patients show a response to this treatment according to the RECIST criteria [62]. Thus, a substantial number of the patients received this toxic combination without benefit. The toxicity of FOLFIRINOX compared to gemcitabine is a serious problem. Identifying non-responders is essential to avoid unnecessary costs and, more importantly, to prevent exposing patients to the adverse events of ineffective and toxic treatment. CTCs could function as biomarkers to address this problem, but studies evaluating this problem have thus been lacking.

A study by Yu et al. investigated a pharmacogenomic (PGx) model to predict treatment response of a PDAC patient to chemotherapy regimens based on the genetic mutations in his CTCs. The authors found that PGx profiling of CTCs can predict treatment response. Additionally, they were able to stratify patients as “sensitive” and “insensitive” for specific chemotherapy regimens, showing the benefits of progression-free and overall survival [63]. It remains unclear, however, whether these models mimic reality in PDAC patients.

#### 3.2.4. Advanced Clinical Utility in Other Entities

Prostate, lung and breast cancer are the pioneer fields in the area of solid cancers in which CTCs detection are used as prognostic and predictive parameters and to evaluate their biologic potential [36,48,64]. These tumors shed higher numbers of CTCs into the blood stream, which makes it more feasible to study their potential [37,65,66]. The number of CTCs before treatment has been described as an independent marker of overall survival in metastatic breast cancer [67] and has also entered the UICC classification as micro-metastases called cM0(i+) [68]. Recently-published results from the SUCCESS trial on over 1000 women with breast cancer showed a CTC positivity of 18.2% two years after chemotherapy. CTCs at baseline and in follow-up were identified as independent markers of an inferior OS [69]. Specific subtypes of CTCs with different expressions of HER2 have been described as clinically relevant [70] and as changing over the course of treatment [71]. Patients with HER2-negative breast cancer but HER2-positive CTCs were investigated in a recent study. In the study, the HER2-targeting trastuzumab did not decrease the detection of HER2-positive CTCs and did not significantly influence survival [72]. In another study by Jakabova et al., the HER2 and ESR status of CTCs differed from the status of the primary tumor. Over the course of treatment, even switches of the HER2 status of the CTCs occurred in some patients [71]. Even larger studies are also needed in this regard. One view for future applications of CTCs in lung cancer was recently given by Palliers et al., who investigated different resistance mutations in the single CTCs of patients with lung cancer resistant to crizotinib, an ALK-rearrangement targeting drug. They found multiple mutations in various genes in ALK-independent pathways and showed the genetic heterogeneity and clinical utility of CTCs to identify therapeutic resistance mutations in ALK-rearranged patients [37]. Single-CTC sequencing may be a unique tool to assess heterogeneous resistance mechanisms and help clinicians to personalize treatment and resistance options in patients with lung cancer but also with other malignancies such as pancreatic cancer.

## 4. Materials and Methods

In September 2019, PubMed, Embase, Medline, Web of Science, Cochrane Library, and Google Scholar were searched using the search terms “Circulating tumor cells, pancreatic cancer, prognosis and diagnosis,” which revealed 145 studies over the last twenty years (1999–2019). This review addresses the role of circulating tumor cells in pancreatic cancer as prognostic and diagnostic tools. The literature search identified articles and reviews addressing this topic. One-hundred-and-two studies were excluded due to their titles and abstracts, wrong tumor entity, or irrelevance in other details. The remaining 43 full text articles were screened for eligibility. 26 were excluded due to non-sufficient data or no relevance to PDAC or CTCs. The remaining 17 articles were analyzed for this review. The criteria identified were the number of patients, country of origin, tumor stage, methods used for the enrichment and detection of CTCs, the detection rate of CTCs in the cohorts, and the outcome parameters (OS, and PFS). Letters, non-English language studies, editorials, and expert opinions were excluded. The bibliographies of included studies were searched for other relevant publications.

## 5. Conclusions and Future Perspectives

PDAC is hardly accessible to tissue biopsy due to its retroperitoneal position. Pre- and intra-therapeutic CTC enrichment and detection can be performed multiple times over a treatment course. The clinical utility of CTCs as prognostic markers and also as risk factors for disease recurrence have been found in patients with PDAC in pilot and prospective midsize studies. However, large-scale studies in PDAC patients with consistent CTC isolation techniques are greatly needed to validate the diagnostic and prognostic potential of CTCs. The predictive value of CTCs has as yet to be sufficiently investigated.

The development from the initiation of mutation to metastatic cancer takes years, giving rise to the potential to interfere before the manifestation of locally advanced or even metastatic tumors. Thus, screening for and the early detection of PDAC in the “windows of opportunity” should be the focus of future trials. Additionally, agreement is needed on a “gold standard” for CTC isolation in PDAC. Due to the EMT features of pancreatic CTCs, this should most likely be a physical enrichment technique followed by a yet-to-be-determined detection.

A single cell analysis of CTCs will prospectively give us more insight into tumor heterogeneity. may reveal particularly aggressive subtypes of CTCs, and may help us to identify treatment targets to fight this devastating disease. A true clinical advantage for patients would be the prevention of invasive carcinoma. Potentially, we will perform more preventive surgery in the future.

## Figures and Tables

**Table 1:** Studies evaluating CTCs in pancreatic ductal adenocarcinoma (PDAC) including survival analysis.

Study	Country	*n*	Tumor Stage	Methodology	CTC Detection Rate *	Outcome	Finding
Soeth et al., 2005 [43]	Germany	172	All stages, mostly IV	Density gradient separation	34%	OS	CTC+: poor OS
Hoffmann et al., 2007 [41]	Germany	37	All stages	Density gradient separation	40%	OS	CTC+: Trend for worse OS
Kurihara et al., 2008 [56]	Japan	26	Stage III and IV	CellSearch^®^	42%	OS	CTC+: sig. worse OS
Sergeant et al., 2011 [42]	Belgium	48	All stages, 40 resectable	RT-PCR (EpCAM)	25% preoperative 65% postoperative	OS	No correlation between CTC and survival
Khoja et al., 2012 [26]	United Kingdom	54	54% non-resectable	ISET^®^; CellSearch^®^	46%/40%	OS; PFS	ISET^®^ detects more CTCs, trend towards decreased OS
De Albuquerque et al., 2012 [57]	Germany	34	All stages (II–IV)	Immunomagnetic (EPCAM)	47%	PFS	CTC + worse PFS
Bidard et al., 2013 [30]	France	79	Non resectable	CellSearch^®^	11%	OS	CTC+ worse OS
Bissolati et al., 2015 [58]	Italy	20	Resectable Stage IIa and b	CellSearch^®^	45%	OS; PFS	No correlation to OS or PFS but slightly higher liver metastasis rate in CTC+
Zhang et al., 2015 [59]	China	22	Stage I–IV, all resectable	Immunomagnetic (CEP 8/ CD45)	15%	OS	CTC+: worse OS
Earl et al., 2015 [60]	Spain	35	Stage II–IV	CellSearch^®^	20%	OS	CTC*: worse OS, almost only metastatic disease
Poruk et al., 2016 [54]	USA	60	All stages	ISET^®^	90%	OS, PFS	Epithelial CTC+: worse OS Epithelial CTC+: earlier recurrence
Gao et al., 2016 [53]	China	25	All stages	CD45 depletion and SE-FISH	I+II: 92.3% III + IV: 83.3%	OS	Patients with lower CTC count better OS than patients with high numbers of CTC
Kulemann et al., 2017 [31]	Germany	58	All stages	ScreenCell^®^	68%	OS	CTC+: Trend to worse OS
Okubo et al., 2017 [52]	Japan	65	III–IV	CellSearch^®^	21%	OS	CTC+ worse OS, more CTC+ in pat. Liver metastases, CTC+ after treatment neg. prong. Factor.
Poruk et al., 2017 [55]	USA	60	All stages mainly I and II	ISET^®^	78%	OS, PFS	CTC labeled with TIC (tumor initiating cell) are predictive of decreased OS and PFS
Gemenetzis et al., 2018 [61]	USA	165	All stages	ISET^®^	95% of resectable patients (*n* = 136)	Identification of mesenchymal-mal and epithelial CTC OS; PFS	Higher CTC counts correlate with earlier recurrence Increase of CTC numbers after neoadjuvant treatment CTC+ correlates with early recurrence and OS in the pretreated group.
Court et al., 2018 [27]	USA	100	All stages 71 localized, 29 metastatic	Nano Velcro Chip enumeration	78%	Identification of occult metastasis; OS	CTC counts correlated with stage and worse OS

* A cut off for CTCs in order to predict prognosis has not been established yet. In different studies, varying amounts of blood and cut offs were used. Maestro et al. detected >2 CTCs/7.5 mL as a prognostically-usable cut off, and others defined this cut off as >1/7.5 mL [26,30]. SeFish: immunostaining-fluorescence in situ hybridization.

## Abbreviations

CTC	Circulating tumor cell	OS	Overall survival
CEC	Circulating epithelial cell	PFS	Progression-free survival
tCTC	Total CTC	EUS	Endoscopic ultrasound
mCTC	Mesenchymal/epithelial CTC	FNA	Fine-needle aspiration
eCTC	Epithelial CTC	CK	Cytokeratin
PDAC	Pancreatic ductal adenocarcinoma	ZEB1	Zinc finger homebox 1
IPMN	Intraductal papillary mucinous neoplasm	SeFish	Immunostaining-fluorescence in situ hybridization
EMT	Epithelial-mesenchymal transition	HER2	Human epidermal growth factor receptor 2
MET	Mesenchymal-epithelial transition	TIC	Tumor initiating cell
